# Intestinal permeability, food antigens and the microbiome: a multifaceted perspective

**DOI:** 10.3389/falgy.2024.1505834

**Published:** 2025-01-09

**Authors:** Francesco Valitutti, Maurizio Mennini, Gianluca Monacelli, Giulia Fagiolari, Marisa Piccirillo, Giovanni Di Nardo, Giuseppe Di Cara

**Affiliations:** ^1^ Department of Medicine and Surgery, Pediatric Unit, University of Perugia, Perugia, Italy; ^2^ European Biomedical Research Institute of Salerno (EBRIS), Salerno, Italy; ^3^ Department of Neurosciences, Mental Health and Sensory Organs (NESMOS), Faculty of Medicine and Psychology, Sapienza University of Rome, Pediatric Unit, Sant'Andrea University Hospital, Rome, Italy

**Keywords:** intestinal permeability, gut barrier, celiac disease, food allergy, eosinophilic gastrointestinal disorders, irritable bowel syndrome

## Abstract

The gut barrier encompasses several interactive, physical, and functional components, such as the gut microbiota, the mucus layer, the epithelial layer and the gut mucosal immunity. All these contribute to homeostasis in a well-regulated manner. Nevertheless, this frail balance might be disrupted for instance by westernized dietary habits, infections, pollution or exposure to antibiotics, thus diminishing protective immunity and leading to the onset of chronic diseases. Several gaps of knowledge still exist as regards this multi-level interaction. In this review we aim to summarize current evidence linking food antigens, microbiota and gut permeability interference in diverse disease conditions such as celiac disease (CeD), non-celiac wheat sensitivity (NCWS), food allergies (FA), eosinophilic gastrointestinal disorder (EOGID) and irritable bowel syndrome (IBS). Specific food elimination diets are recommended for CeD, NCWS, FA and in some cases for EOGID. Undoubtfully, each of these conditions is very different and quite unique, albeit food antigens/compounds, intestinal permeability and specific microbiota signatures orchestrate immune response and decide clinical outcomes for all of them.

## Introduction

The gut barrier is a selective filter for the gastrointestinal (GI) tract, which is exposed to a wide range of external antigens, including food, microbes, and toxins (1). This barrier encompasses several interactive, physical, and functional components: (1) the gut microbiota; (2) the mucus layer; (3) the epithelial layer; (4) the mucosal immunity. Each unit is key in shielding the host from harmful ingested antigens. The microbial barrier, or gut microbiota, consists of trillions of microorganisms and acts as a significant protective shield against pathogens. It contributes to host health, facilitating nutrient absorption, metabolism, and immune regulation. This multitude of microorganisms can induce immune cell maturation and appropriate response for host defense (2). The trajectories of microbiota composition vary during life. Neonatal gut microbiota is composed predominantly by anaerobic bacteria, with a greater abundance of Bifidobacteria in those who are breastfed (3).

After the first few years of life, the microbiota becomes more stable and “adult-like” in its composition (4), but it could be still perturbated by dietary choices, hormones, antibiotics and pollution (5–8). The resistance and resilience of the gut microbiota depends on various reasons: - diversity; - functional redundancy;—adaptive capacity;—intermicrobial interactions;—host immunity;—diet;—environmental consistency, which means the stability of nutrients, pH and oxygen level;—gut habitat complexity, i.e., having different anatomical and functional units such as folds and crypts allowing more stable bacterial reservoirs (9).

The mucus layer consists of an extracellular coat located outside the intestinal epithelial cells (IECs). Mucus is composed of mucins, principally Muc17 in the small intestine and Muc2 in the colon, which are glycoproteins overlapping each other in a net-like fashion (10).

Epithelial cells lining the intestine are vital for maintaining a tight interplay between the luminal environment and the host. This epithelium comprises several cell types derived from stem cells that constantly replenish differentiated intestinal epithelial cells (IECs), eventually shedding into the lumen (11). IEC subsets are broadly categorized into absorptive and secretory epithelial lineages. Most epithelial cells are absorptive enterocytes, albeit their active involvement in mucosal immunology needs to be further elucidated. Secretory IECs include: -enteroendocrine cells; -Paneth cells, which sustain stem cells and secrete antimicrobial peptides; -goblet cells, which deploy mucus production; -tuft cells, which detect luminal signals and promote type 2 immunity (12, 13). Differently from absorptive and secretory cells, Microfold (M) cells constitute a unique IEC subset that allow the trans-cytoplasmatic transportation of luminal antigens to immune cells into the lamina propria (14).

Altogether, epithelial cells throughout the intestinal tract orchestrate nutrient absorption, pathogen defense, and immune regulation. Antigens that reach the intestinal epithelium can be transported by different routes depending on their size and solubility. Soluble antigens are absorbed by enterocytes and transported mainly via the transcellular route. Under homeostatic conditions tight junctions between enterocytes prevent the paracellular passing of antigens, preserving the cell polarity necessary for directional functions. Several proteins, such as angulins, occludins, and claudins constitute these junctions (15).

**Figure 1:**
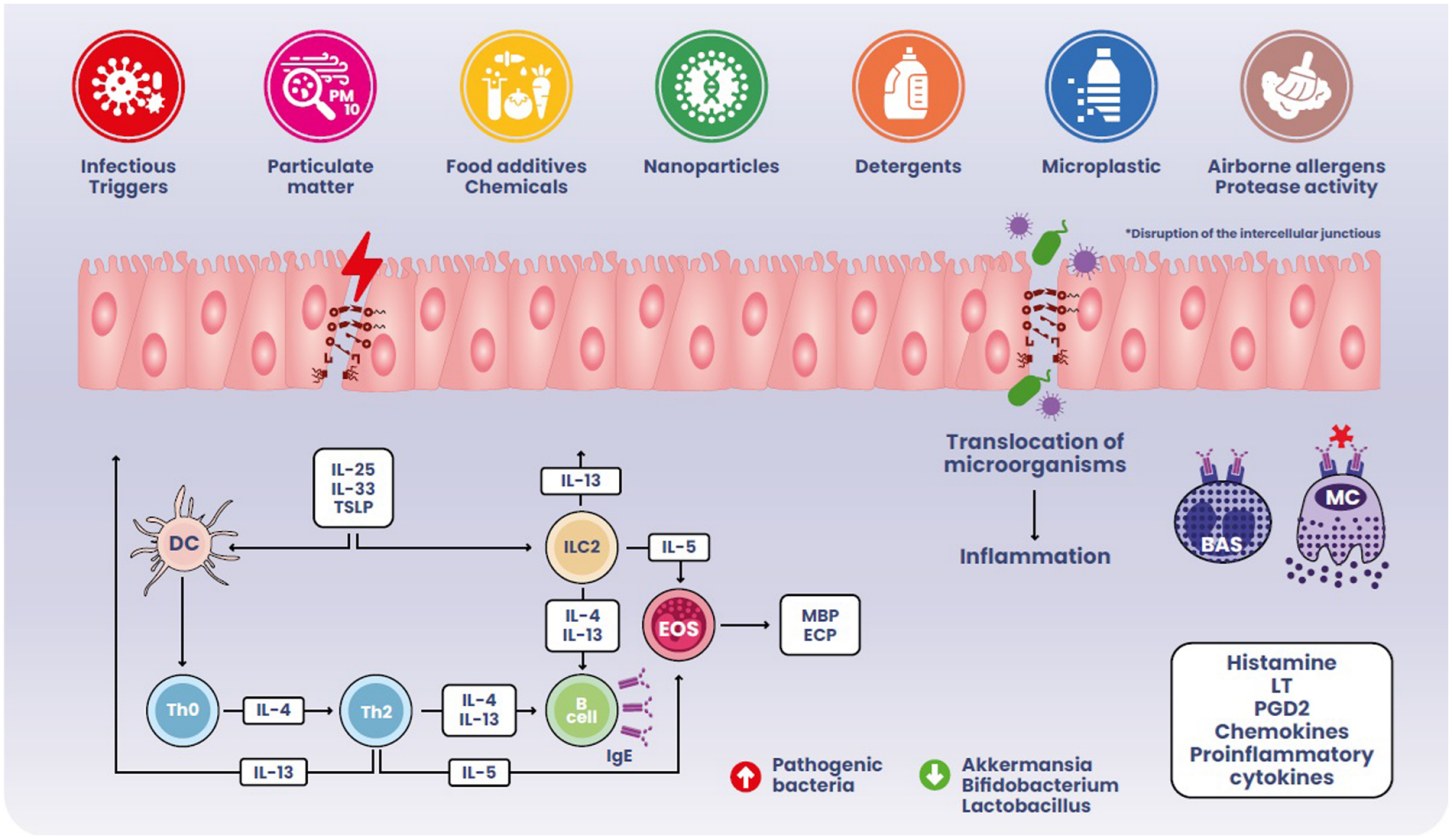
Complex interplay among environmental factors, epithelium and food allergy response.

The presence of numerous foreign antigens in the intestinal lumen implies the accumulation of immune cell populations within the GI tract. Nonetheless, immune cell subsets in the intestine coexist to uphold homeostasis towards various stimuli in a well-regulated manner. Disrupting the mechanisms underlying this balance diminishes protective immunity and leads to the onset of chronic inflammatory diseases as exemplified in Figure 1.

In this review we aim to summarize current evidence linking food antigens, intestinal microbiota and gut permeability interference in diverse disease conditions such as celiac disease (CeD), non-celiac wheat sensitivity (NCWS), food allergies (FA), eosinophilic gastrointestinal disorder (EOGID) and irritable bowel syndrome (IBS).

## Food antigens clashing into the barrier

### Gluten, celiac disease and non-celiac wheat sensitivity

Celiac disease (CeD) is a systemic immunological disorder triggered by the intake of gluten and related prolamins in genetically at-risk individuals, i.e., those bearing HLA-DQ2 or HLA-DQ8 haplotypes (16). It presents with a wide range of clinical symptoms, the presence of CeD-specific antibodies and immune-mediated intestinal damage. While CeD can occur at any age, most at-risk individuals are diagnosed during their early childhood, typically within the first five years, as shown also by several prospective studies (17–19). Due to its diverse systemic and malabsorptive symptoms, CeD is often described as a “clinical chameleon,” which can pose challenges for primary care practitioners (20). Currently, many individuals with CeD experience few symptoms or predominantly exhibit extraintestinal issues, while only a minority manifests the “classical” signs of malabsorption characterized by weight loss, failure to thrive, and persistent diarrhea (21). The only effective treatment for CeD is a strict gluten-free diet (GFD), albeit several investigational drugs are in phase 2 clinical trials for non-dietary/complementary management strategies (22).

In CeD patients, gluten-dependent increased intestinal permeability is a paradigm of the disease, priming and perpetrating intestinal damage on a gluten-containing diet. Zonulin is a gastrointestinal paracrine hormone that, together with other growth factors, negatively affects intercellular integrity by disassembling tight junctions (23).

Among the various potential stimuli in the intestinal lumen that can induce zonulin release, small intestinal exposure to microorganisms and gluten have been identified as the most potent triggers (24, 25). From an evolutionary perspective, the zonulin-induced opening of the paracellular route might act as an ultimate defensive process that washes out microorganisms, working alongside other similar mechanisms, such as the IL-22-mediated modifications of intestinal permeability against bacteria (26).

Gliadins are gluten-complex proteins usually rich in prolines and glutamines, which are not digested by intestinal enzymes. Few peptides that residue from partial digestion of gliadins impact intestinal barrier function by triggering the release of zonulin upon interaction with the chemokine receptor CXCR3 (27). Gliadin thus rapidly and temporarily enhances zonulin-dependent paracellular permeability of the gut, regardless of disease status (28). Notably, once the GFD is commenced, the intestinal paracellular permeability is switched off (29).

Genetic studies have supported the role of the paracellular pathway in gluten transport within the lamina propria, which have found a connection between specific tight junction-related genes and CeD (30, 31). In CeD, the loss of barrier function is the first step towards the loss of tolerance to gluten and the consequent damage to the intestinal mucosa. This is witnessed by the fact that healthy first-degree relatives of patients with CeD might present with increased intestinal permeability (32). A preliminary study from the CD-GEMM (Celiac Disease Genomic Environmental Microbiome and Metabolomic Study) prospective cohort suggests that in infancy the onset of CeD is preceded by an increase of zonulin levels months before the disease manifests. This rise is associated with a medical history of multiple courses of antibiotics, thus linking microbiota disturbances to the subsequent loss of immune tolerance towards gluten (33). In addition, infections by enteroviruses and rotaviruses have been accounted as putative triggers for CeD onset in longitudinal studies (34, 35). Barrier breach and antigen molecular mimicry could be hypothesized as underling mechanisms preceding disease inception (36, 37).

Within the spectrum of gluten-dependent disorders, another condition is the so called non-coeliac wheat sensitivity (NCWS), currently defined as a wide set of intestinal and extraintestinal symptoms after gluten consumption in subjects for whom CeD and wheat allergy have been thoroughly excluded (38).

However, with except of symptoms such as brain fog, fatigue, and joint pain, several features of NCWS overlap with functional GI disorders, particularly IBS, thus posing a challenging task for physicians who deal with these patients. To discriminate the two conditions, Barbaro et al. have demonstrated that increased serum levels of zonulin are more frequently present in NCWS rather than in IBS, therefore highlighting within a clinical framework the role of intestinal permeability in this condition (39). In addition, Ahmed et al. very recently reported that the presence of conventional anti-gliadin antibodies (AGA) is strongly predictive of NCWS among IBS patients (40). Since AGA have been previously associated with increased intestinal permeability (41), this evidence reinforces the role of “leaky” gut in NCWS.

Another *in vivo* study highlighted intestinal epithelial defects at fluorescein-based confocal microscopy a few minutes after wheat exposure in several patients with IBS-like symptoms. This was linked to a heightened expression of the pore-forming protein claudin-2, thus suggesting that a leaky gut seems to coexist with NCWS misdiagnosed as IBS (42). Furthermore, another group has reported elevated serum levels of lipopolysaccharide-binding protein in patients with NCWS, advocating an increased passage of microbial components from the lumen to the systemic circulation. Notably, this phenomenon seems to revert on a GFD (43).

Notwithstanding these data from pre-clinical and clinical studies, it should be considered that other translational studies on this topic have shown contrasting results.

For example, Sapone et al. showed similar permeability parameters in the small bowel of NCWS individuals and patients with dyspepsia using the lactulose/mannitol ratio (44). Another study measured the increase in permeability assessed as modification of transepithelial electrical resistance from *ex vivo* intestinal biopsies after exposure to gliadin: no increase was found in those from NCWS subjects compared with those from healthy controls (45). Nevertheless, it should be considered that both these studies were affected by small sample size and that both methods used to determine intestinal permeability are pretty variable.

Exposure to wheat components other than gluten are currently under scrutiny as possible etiology drivers for NCWS. Amylase-trypsin inhibitors (ATIs) are water-soluble globulins from wheat that play a role in grain maturation, carbohydrate storage and defense from parasites. They are major stimulators of innate immune cells but initially they were not considered to directly affect intestinal permeability (46).

However, in a model of specific pathogen-free mice lacking Toll-like receptors, a Canadian group has reported that ATIs can increase intestinal permeability assessed by Ussing chambers (47). Of note, tissue conductance was ameliorated when specific strains of Lactobacilli expressing enzymes for digesting ATIs were provided in the medium. This experiment has shown an exciting attempt to counteract barrier dysfunction in gluten-related disorders. Nevertheless, for the time being evidence has been scant regarding the possibility of modulating intestinal permeability through other probiotic interventions.

There is extensive literature on numerous strains of Lactobacilli and Bifidobacteria possessing proteolytic enzyme machinery for gluten. Still, these have been tested mainly for improving clinical outcomes, and they were not assessed as regards peptide residual interference on intestinal permeability (48). Fewer studies focused on probiotics and intestinal permeability in the setting of experimental models for CeD. An interesting study from Lamas et al. highlighted in NOD/DQ8 mice the indirect action of L. reuteri that reduced intestinal permeability by enhancing the tryptophan-derivates pathways (49). Moreover, on Caco-2 cells, a specific strain of Bifidobacterium (B. lactis) could inhibit the gliadin-induced increase in epithelial permeability, and this effect of the strain was clearly dose-dependent (50). Another preclinical study on L. rhamnosus GG showed that this latter strain sustained the expression of the intercellular junction proteins, thus contrasting the increase in intestinal permeability in the context of murine enteropathy induced by pepsin-trypsin-digested gliadins (51).

### Food allergens: food allergy and eosinophilic GI disease

Food allergy (FA) is defined as “an adverse health effect arising from a specific immune response that occurs reproducibly on exposure to a given food”. FA is a growing public health concern, with increasing prevalence in Western countries. It currently affects 8% of children and 3% of adults, with prevalence rising every year and significantly varying depending on the method of assessment (self-reported FA vs. gold standard oral food challenge) (52–54). According to the pathophysiologic immune mechanisms, it can be classified into IgE-mediated, non-IgE-mediated (cell-mediated) or mixed (IgE and cell-mediated) (55, 56).

Post-translational modifications of antigens, including processes such as glycosylation, phosphorylation, acetylation and hydroxylation, may alter the allergenic potential of food proteins (57). These modifications may enhance protein stability, modify epitopes that immune cells recognize or affect how proteins are processed and presented to the immune system. As a result, the same protein can elicit different immune responses depending on its post-translationally modified forms, contributing to the variability observed in allergic reactions among individuals. For example, glycosylation of casein hinders its full digestion, therefore its allergenicity is enhanced (58). As regards peanut allergy, boiled vs. roasted peanut proteins show a marked difference in their ability to trigger IgE response. Boiling tends to denature some of the proteins responsible for allergic reactions and this can lead to a less severe allergic response in sensitive individuals (59). In contrast, roasting provokes the Maillard reaction, which in its early phases is a glycation of proteins; glycated peanut proteins are more likely to trigger allergic reactions due to a preferential recognition by dendritic cells (60).

Although genetic factors play a role, FAs are increasing in prevalence at a higher rate that cannot be explained only by genetics (61).

It has been known for years that many environmental factors are associated with the development of FAs, such as caesarean section, formula feeding, early antibiotic exposure, not having siblings or pets; nevertheless, the connection between epidemiological data and the underlying immune mechanism remains elusive. This gap is being filled by increasing evidence about gut microbiota as one of the main actors in the complex mechanism of sensitization. Indeed, the previous mentioned epidemiological factors have been shown as culprits in the development of dysbiosis (62–67).

The gut microbiota, which consists of trillions of microorganisms including bacteria, viruses, fungi, and archaea, plays a vital role in maintaining immune homeostasis and protecting the epithelial barrier.

Human milk microbiota and the birth canal in vaginal delivery are the two main sources through which infants can acquire microbes colonizing gut lumen. Moreover, children ingest every day with breast milk an amount of bacteria ranging between 1 × 10^5^ and 1 × 10^7^, which is almost 30% of whole infant bacteria pool (68).

Human milk could play a pivotal role in preventing or promoting allergy development by modulating gut microbiota biodiversity and gut barrier function due to the activity of molecules such as milk oligosaccharides, glycomacropeptides, lactoferrin, defensins, and other metabolites (69). Human milk oligosaccharides (HMO) are non-digestible oligosaccharides having a very important probiotic role: they inhibit bacterial adhesion to intestinal mucosa surface and prevent colonization of certain bacteria strains (70). Casein glycomacropeptides, defensins and tryptophan metabolites have also shown specific antimicrobial properties (71). Finally, lactoferrin, a protein that binds and separates iron from bacterial pathogens, has both antiviral and antibacterial activities (72).

The composition of human milk microbiota may be influenced by many factors such as stage of lactation, maternal body mass index (BMI), age and diet, geographical location, socioeconomic status, use of antibiotics and probiotics during pregnancy (73–77).

As regards specific microbiota signatures and atopy, Lachnospiraceae have shown to be well represented in children allergic to cow's milk (78); moreover, Canadian Health Infant longitudinal development (CHILD) study has shown that a reduction of genera such as Lachnospira, Veilonella, Faecalibacterium and Rothia, and an abundance of Clostridium difficile and Staphylococcus aureus are linked with asthma development later in life (79).

An early gut colonization by Bacteroides fragilis expressing polysaccharide A could inhibit Treg proliferation, therefore predisposing towards allergy (80).

It has been also highlighted that early colonization with Clostridium genera is related to an increased IL-22 secretion by group-3 innate lymphoid cell and T-helper 17 and specific IgE production in children with FA (81).

The importance of microbiota-host relationship in allergy is witnessed by murine models of germ-free mice displaying elevated blood levels of IgE. Instead, after microbial colonization which starts between birth and the first week, IgE production is completely inhibited (82).

Among the important functions that intestinal microbiota plays in the host, its contribution to immune tolerance for orally administered antigens is key. Oral tolerance is a state of systemic unresponsiveness that should be the default response to food antigens in the GI tract. This is an active process that begins with the uptake of potential food antigens by immune cells of the gut-associated lymphoid tissue (GALT) in the small intestine (83). Antigens are then degraded into small peptides inside vesicles, loaded into MHC-II in endosomes and released to be taken up by CD103 + dendritic cells (84, 85). Antigens with resistance to proteolysis can reach the basolateral membrane in an intact form (86). M cells can endocytose antigens and myeloid cells such as dendritic cells and/or macrophages may directly capture potential food antigens from the gut lumen by extending a process through a tight junction (periscoping behaviour) or by extending a process through a transcellular pore in an M cell (87, 88). These dendritic cells can then migrate from the lamina propria to the draining lymph nodes where the dendritic cells express transforming growth factor-β (TGFβ) and retinoic acid, inducing naive T cells to differentiate into regulatory T (Treg) cells; therefore, the cell-mediated trafficking of antigen to the secondary lymphoid tissue promotes the establishment of tolerance (89, 90). Tregs regulate the immune response by producing inhibitory mediators, such as IL-10 and TGF-β (91).

Dendritic cells also imprint GI homing capacity, allowing the recently primed Tregs to home back to the lamina propria where they interact with macrophages that produce IL-10 and expand. In addition, anergy and T cell depletion have also been shown as mechanisms of oral tolerance, with anergy referring to T-cell unresponsiveness to the antigen and depletion to the apoptosis of antigen-specific T cells. These two mechanisms cooperate depending on the dose of antigen exposure: high dose antigen exposure induces anergy or depletion while low dose antigen leads to induction of Tregs (92).

The microbiota plays a key role in the development of oral tolerance by multiple ways. Intestinal bacteria stimulate the production of mucus glycoproteins, sealing the intestinal barrier and protecting the epithelium from the growth of pathogenetic bacteria. In addition, they have a role in the modulation of Th1/Th2 response in favor of a tolerogenic immune response and in the activation of Tregs: these events are mediated by metabolites such as butyrate, a short-chain fatty acid (SCFA) produced by the fermentation of dietary fiber in the colon, which has a strong immunoregulatory effect (93, 94).

On the other hand, several studies in mice have demonstrated an association between compositional and functional changes of the gut microbiota, also known as dysbiosis, and development and progression of FA (95, 96). Early studies showed that germ-free mice were unable to achieve oral tolerance to food allergens (97). Recent studies have further shown that transferring gut microbiota from patients with FA to germ-free mice can transmit susceptibility to FA (98). Conversely, germ-free mice colonized with bacteria from healthy infants were protected against anaphylactic responses to a cow's milk allergen (99). Certain microbial orders, including Clostridiales and Lactobacillales, have been related to suppression of FA in mouse studies (100). Similarly, Bacteroidales and Enterobacteriales have been described to have both beneficial and detrimental effects with regards to FA (101, 102).

The hypothesis on gut dysbiosis preceding FA relies on several basic science and clinical studies showing proinflammatory microbiota changes nurturing a chronic, low-grade, inflammation (103–120). Moreover, intestinal dysbiosis cooperates with a leaky gut barrier in FA pathogenesis (121–123). External factors (particularly the consumption of ultra-processed foods, but also other dietary factors, infections, inflammatory bowel diseases etc.) can influence both the gut microbiota and epithelial barriers leading to a pro-inflammatory milieu: when the epithelial barrier is damaged, antigens may cross freely, causing the release of pro-inflammatory epithelial-derived molecules like IL-25, IL-33 and thymic stromal lymphopoietin (TSLP), which promote T-naïve cell differentiation into Th2 cells, IgE class-switching, and tissue accumulation of mast cells and eosinophils (124). After sensitization to a food allergen, allergen-specific IgE antibodies bind to basophils and mast cells surface IgE receptors. A subsequent exposure to the allergen can cause these cells to immediately release histamine and other proinflammatory mediators, such as leukotrienes and prostaglandins, that lead to tissue inflammation and recruitment of inflammatory cells, worsening gut permeability and amplifying type-2 inflammation (125). Several studies have identified a mutual link between increased intestinal permeability and FA: not only the establishment of FA is favored by a leaky intestinal barrier, but also the allergic response further impacts intestinal permeability allowing the translocation of allergenic molecules (126–128).

Complementary food introduction is known to influence gut microbiota composition and future health outcomes. The first 1,000 days of life indeed seem to represent the critical window of opportunity for microbiota modulation. A delayed introduction of solid food could cause a lag in microbial maturation increasing susceptibility to allergies and obesity (129). On the other hand, an earlier introduction could expose infants to potential pathogens and allergens. Therefore, the timing of solid food introduction should be balanced between these risks and benefits (130, 131). Particularly, a meta-analysis on the timing of introduction of egg and peanut in infant diet showed indication to introduce egg at age 4–6 months and peanut at age 4–11 months (in an age-appropriate form to avoid risk of inhalation), as this behavior was associated with reduced egg and peanut allergy (132). Notably, this is strikingly conflicting with the previous recommendation to delay the introduction of allergenic foods to the infant diet to prevent sensitization (133).

An earlier introduction of multiple allergenic foods was associated with reduced IgE-mediated allergy, consistent with the findings of the Preventing Atopic Dermatitis and Allergies in Children (PreventADALL) trial. Even though safety data were generally reassuring, their findings were limited by high rates of withdrawal from the intervention (134).

Breast-feeding and diet diversity promote a healthy microbiota not only during the weaning period, but also over the whole critical time of the two first years of life. After this period, the gut microbiome tends to acquire an adult-like configuration with distinct microbial community composition and functions. A varied and healthy diet appears to confer a positive modulation of gut microbiota: a significant association between higher diet diversity and a lower prevalence of parent-reported, doctor diagnosed food allergy has been demonstrated (135).

The immunologic basis explaining how diet diversity potentially affects allergy outcomes may be mediated by the induction of tolerance mechanisms including T and B regulatory cells, immune regulatory cytokines and suppressed IgE antibodies.

A more diverse diet may indirectly affect tolerance development via an effect on the microbiota as increased diet diversity leads to increased microbial diversity in infants at weaning. Moreover, a more diverse diet may also lead to exposure to different food antigens that impact on the development of immune tolerance, though this may be “low dose” exposure, supporting recent randomized controlled trials regarding early allergen introduction (136).

Though there is a need to harmonize study methods and define diet diversity for studying adequate dietary intake and allergy outcomes, diet seems to be a modifiable factor that can be used to prevent or manage allergic disease (137).

Restrictive dietary regimens of children with specific diseases (FA, inborn errors of metabolism, CeD, etc.) may alter the gut microbiota triggering the overgrowth of intestinal inflammation. Elimination diets that decrease SCFA-producing bacteria, such as the gluten-free diet, phenylketonuria diet, and ketogenic diets and those with overall low consumption of a plant-based products may have negative effects on the microbiota (138–141).

Despite a significant impact on some pre-clinical and clinical aspects, current evidence does still not support a clear recommendation on the use of probiotics in the prevention or treatment of FA. Clear information on the specific strain, dosage, and adequate duration of therapy is lacking. Moreover, future strain-specific studies should ideally take into account that gut microbiota also reflects the integrity of the gut barrier and any supplementation approach should contextually aim for its restoration. A summary of specific bacteria/probiotics and their impact on food allergies is presented in Table 1.

**Table 1:** Literature summary of specific bacteria/probiotics and their impact on food allergies.

Species/Strain	Type of study	Outcome	Year	No. of patients	Author	Study PMID
L. rhamnosus GG (LGG)	- *in vivo* - Placebo-controlled-prospective randomized controlled study	Suppression and prevention of allergic response	2019	na	Fu et al.	PMID 30660420
			2001	132	Kalliomäki et al.	PMID 11297958
L. plantarum JC7	- *in vivo* - *in vivo*	Microbiota/barrier restoration in food allergy	2022	na	Duan et al.	PMID 36194269
			2021	na	Jiang et al.	PMID 34606539
L. plantarum HM-22						
L. acidophilus KLDS 1.0738	- *in vivo* - *in vitro*	Inhibition of allergic response and suppression of allergic pathways	2019	na	Ni et al.	PMID 31477403
			2021	na	Li et al.	PMID 33990976
L. casei	- *in vivo* - Randomized, double-blind, placebo-controlled, parallel-group study	- Alleviation of tropomyosin-induced food allergy - Benefits on atopic dermatitis for children with CMP allergy	2020	na	Fu et al.	PMID 32243079
L. casei LOCK 0918			2021	151	Cukrowska et al.	PMID 33916192
B. longum longum 51A	- *in vivo* - *in vivo*	- Reduction of allergic inflammation	2021	na	Santos et al.	PMID 34558015
			2021	na	Pyclik et al.	PMID 34354710
B. longum longum CCM 7952						
B. bifidum TMC 3115	- Randomized double-blind control trial - Multicenter, randomized, and controlled clinical trial	- Improving of anti-inflammatory responses - Reduced risk of gut dysbiosis	2020	256	Jing et al.	PMID 34741472
			2024	413	Bellomo et al.	PMID 38930475
B. infantis 14.518	- *in vivo*	- Alleviation of Tropomyosin-induced allergic responses	2017	na	Fu et al.	PMID 29176981
A. muciniphila	- *in vivo* - *in vivo*	- Detrimental effect in case of fibre deprivation - Protective effect to ovalbumin food allergy	2023	na	Parrish et al.	PMID 37696941
A. muciniphila BAA-835			2023	na	Miranda et al.	PMID 37097372
B. coagulans 09.712	- *in vivo*	- Ameliorates shrimp tropomyosin induced allergic response via suppression of mTOR signalling	2017	na	Fu et al.	PMID 28512288
C. butyricum CGMCC0313-1	- *in vivo* - *in vivo* - *in vivo*	- Reduction of beta-lactoglobulin-induced intestinal anaphylaxis - Reduction of ovalbumin-induced allergic airway inflammation - Strengthening of gut barrier function And attenuation of disease in models of colitis and allergic diarrhoea	2017	na	Zhang et al.	PMID 28250847
			2017	na	Juan et al.	PMID 28122397
			2018	na	Wang et al.	PMID 30014710
C. butyricum CGMCC 7281						
L. acidophilus CGMCC 7282 plus C. butyricum CGMCC 7281						
Clostridiales	- Cohort study - Prospective case-control follow-up study	- Increased Bacteroidales and reduced clostridiales in allergic adults - Higher total bacterial and anaerobic counts in cow's milk protein allergic children compared with healthy children	2015	1879	Hua et al.	PMID 26870828
Bifidobacteria			2009	92	Thompson-Chagoyan et al.	PMID 19889194

Eosinophilic gastrointestinal diseases (EGIDs) are a group of chronic inflammatory disorders characterized by eosinophilic infiltration into various segments of the GI tract, including eosinophilic esophagitis (EoE), eosinophilic gastritis (EoG), and eosinophilic colitis (EoC) (142). The pathophysiology of these conditions is multifactorial, involving complex interactions between environmental triggers, such as food antigens, immune responses, and alterations in the epithelial barrier and the gut microbiota (143, 144). Recent studies have provided both pre-clinical and clinical evidence that these diseases are associated with significant changes in gut permeability, membrane integrity, and microbita composition (145). These alterations appear to play a pivotal role in disease progression and symptom manifestation.

One of the critical components of EGIDs is the dysfunction of the intestinal epithelial barrier, which is often referred to the aforementioned leaky gut. This term describes the increased permeability of the gut lining. In EGIDs, intestinal barrier is compromised, leading to increased intestinal permeability (146). Pre-clinical models have demonstrated that this increased permeability may facilitate the translocation of food antigens and bacterial products into the lamina propria, thereby triggering or exacerbating local immune responses. Specifically, it has been shown that eosinophils, key effector cells in these diseases, are recruited to the gut in response to antigen exposure, where they release pro-inflammatory mediators such as cytokines, chemokines, and granule proteins (including major basic protein and eosinophil-derived neurotoxin), which further disrupt epithelial integrity and amplify the inflammatory response.

Clinically, patients with EGIDs exhibit a range of symptoms that are closely linked to this compromised epithelial barrier. Small bowel permeability is overall increased in patients with active EoE, and is normal in patients with EoE in remission when compared to healthy controls (147). In eosinophilic gastritis, patients may present with abdominal pain, nausea, vomiting, and malnutrition, symptoms that are also likely exacerbated by a dysfunctional epithelial barrier that allows for abnormal exposure to luminal antigens and microbial products. Endoscopic evaluations in these patients often reveal mucosal erythema, edema, and furrowing, further evidence of the inflammation and tissue damage caused by increased permeability (148).

A growing body of literature also highlights the significant role of the microbiome in the pathogenesis of EGIDs. Alterations in the composition and diversity of the gut microbiota, known as dysbiosis, have been also implicated in EGIDs. In the context of EoE, for instance, several studies have reported significant differences in the esophageal microbiota of patients with the disease compared to healthy controls. Specifically, there is evidence of a relative abundance of Streptococcus species in the esophagus of EoE patients, which may contribute to disease pathogenesis by promoting a pro-inflammatory environment that favors eosinophil recruitment and activation (149).

The increase in permeability is believed to result from a disruption in the regulation of tight junction proteins, specifically claudin 1 (CLDN1) and zonula occludens 1 (ZO-1) (150). However, it is possible that the compromised mucosal integrity observed in EoE may be secondary to byproducts of the inflammatory infiltrate, suggesting that it could be a consequence rather than a cause of the inflammation. Previous research has indicated that the inflammatory cytokines associated with EoE, including IL-13 and IL-5, have the ability to downregulate key barrier proteins such as desmoglein (DSG1) and filaggrin (FLG) (151, 152). Interestingly, in the context of atopic dermatitis, similar barrier dysfunctions have been observed not only in the skin but also in the small intestine (153). This raises an important question: is the esophagus the only site of allergen penetration in EoE, or could allergens also enter through the small intestine, potentially triggering or exacerbating inflammation in the esophagus? The current evidence on whether the duodenum plays a role in EoE is conflicting. One study in adults found increased small intestinal permeability (147), while another study in children did not show such findings (154). Regardless, the integrity of the mucosa in both the esophagus and possibly the small intestine may be crucial to understanding the disease's pathology, and restoring this integrity may be key to achieving complete histological remission. Elemental diet studies, which exclude food allergens, have demonstrated significant clinical and pathological improvements in EoE (155, 156). However, the impact of eliminating food allergens on mucosal integrity has not yet been explored in these patients. In addition to changes in microbial composition, recent research has suggested that the function of the microbiota may also be altered in EGIDs (157). The gut microbiome is known to produce a variety of metabolites that influence both local and systemic immune responses. For example, SCFA have been shown to have anti-inflammatory effects and to promote the integrity of the epithelial barrier (158). However, in patients with EGIDs, there may be a reduction in the production of these beneficial metabolites, leading to a dysregulated immune response and further compromising barrier function. This hypothesis is supported by a study demonstrating that the dominant taxa in patients with EGIDs was increased (Streptococcus in esophagus; Prevotella in stomach) (149). Specific taxa were associated with active disease for both EoE (Streptococcus, Gemella) and EoG (Leptotrichia), although highly individualized communities likely impacted statistical testing. Stool analyses did not correlate with bacterial communities found in mucosal biopsy samples and was similar in patients and controls. Therefore, further study is needed to determine if therapeutic interventions contribute to the observed community differences.

Nevertheless, pre-clinical models of EGIDs have provided valuable insights into the mechanisms by which microbiota alterations contribute to disease pathogenesis (159). In mouse models of EoE, for example, it has been shown that antibiotic treatment, which disrupts the gut microbiota, can exacerbate disease symptoms and lead to increased eosinophilic infiltration of the esophagus. Conversely, the administration of probiotics or microbiota-targeted therapies has been found to reduce eosinophil levels and improve epithelial barrier function, suggesting that modulating the gut microbiota may be a promising therapeutic strategy for patients with EGIDs (160, 161).

The clinical implications of these findings are striking. Current treatment options for EGIDs primarily focus on suppressing the immune response and reducing eosinophilic inflammation through the use of corticosteroids, biologic agents (such as anti-IL-5 and anti-IL-4Rα therapies), and dietary interventions (such as elimination diets that remove suspected food triggers). However, emerging evidence suggests that targeting the gut microbiota may represent a novel therapeutic approach for these diseases (162). For example, clinical trials are currently underway to evaluate the efficacy of fecal microbiota transplantation (FMT) and other microbiota-targeted therapies in patients with EoE and other EGIDs. These therapies aim to restore a healthy microbial balance in the gut, thereby improving epithelial barrier function and reducing inflammation.

The need for further research is evident, particularly in exploring how dietary interventions and microbiota-modulating therapies affect barrier integrity and inflammation. Understanding the mechanisms by which allergens and microbial products interact with the immune system in these diseases will be crucial for developing more effective, targeted treatments aimed at both preventing eosinophil infiltration and restoring mucosal health.

## Other food components as barrier disruptors: emulsifiers, plastic nanoparticles and advanced glycation end products

A higher consumption of processed foods in Western and Westernized countries has unfortunately been driven by the increase in productivity in modern societies. These foods are rich in food additives such as emulsifiers, plastic nanoparticles and AGEs (advanced glycation end products) which can all pose risks for human health (163).

Emulsifiers play a very important role in industrial food production, helping to combine immiscible ingredients such as oil and water, and can be commonly found in many processed foods. They are widely used in industrial processed food as they optimize food properties and help to exalt appearance, texture and flavor. Main food emulsifiers include guar gum (E412) found in cheese and other dairy products, lecithin (E32) commonly used in chocolate, xanthan gum (E415) usually found in mayonnaise, carrageenan (E407) found in ice creams/desserts and polysorbates (E432-436) found in ice cream, cakes and oils (164).

Polysorbate—60, polysorbate—80, carboxymethylcellulose (CMC), glyceryl monolaurate and carrageenan are mainly studied and, as shown *in vitro* and in animal models, they are known for their detrimental effect on human health and intestinal inflammation due to their impact on gut microbiota and gut permeability (165–177).

Emulsifiers may determine gut dysbiosis and reduce biodiversity of gut microbiota: many studies have proven an emulsifier-driven reduction of Lactobacillales and Clostridiales such as Faecalibacterium genera and specifically Faecalibacterium prausnitzii in inflammatory bowel disease (IBD); on the contrary, an increase of other specific commensals such as Escherichia, Roseburia, Bradyrhizobium and Turicibacter genera have been also described on a emulsifier-rich diet (178).

As shown in murine and human models, mucus layer thickness was reduced upon exposure to CMC and polysorbate 80, common emulsifiers used in foods, and there were also an increased bacterial penetration of the mucus and a shift to proinflammatory microbiota composition (179, 180).

It has been demonstrated that polysorbate 80 could increase the viscosity of mucus, due to smaller pores in mucus layer and those changes could accelerate movement of some bacteria as Escherichia Coli and modify interaction with other bacteria (181).

These changes in intestinal barrier could determine an increased permeability and a greater bacterial translocation. In fact, it has also been shown that exposure to carrageenan changed location of zonula occludens (Z0-1) from peripheral to more central position in cell membrane and altered actine filaments (182). Carrageenan also causes non-reversible modification in microbiota composition, bacterial density and enhancement of pro-inflammatory pathways (168, 183). These mechanisms could be crucial for determining intestinal inflammation at the onset or perpetrating IBDs.

In addition, CMC and polysorbate 80 also have a pro-inflammatory effects, as they induce an increased intestinal production of tumor necrosis factor (TNF)-α, IL-1 β, IL-6, IL-8 and activated B cells unleashed by Toll-like Receptor-4 (TLR4) (184).

As regards the possible involvement of emulsifiers in Th2-type adaptive immunity, in murine models a dietary exposure to CMC and polysorbate 80 increased IL-4 and IL-5 gene expression; specifically, the latter upregulated gene involved in histamine synthesis, IL-4, IL-5, IL-13, IL-33 and other genes involved in mast cell activation (185).

Another important role in barrier disfunction could be played by plastic nanoparticles (NP), which are commonly found in human food, due to an increased release of plastic waste in the environment. Among plastic particles, those with less than 100 nm are more easily internalized by enterocytes through endocytosis, avoiding tight junction defense system. Their role, especially for long term exposure, is still to be completely defined as a health risk factor, both alone or as a cofactor when epithelial barrier presents a dysfunction. While several inorganic plant-derived NP may reduce diversity of microbiota (186), other NP might interfere with immune system and gut-brain axis, through an exacerbation of oxidative stress or an increase in gut permeability (187, 188).

A summary of studies on specific food additives and their impact on gut permeability/inflammation/microbiota is shown in Table 2.

**Table 2:** Literature summary of specific food additives/nanoplastic compounds and their impact on gut permeability/inflammation/microbiome.

Food Additive	Type of Study/Model	Outcome	Year	Author	Study (PMID)
Carboxymethylcellulose	Murine model	Increased inflammation levels and colitis driven by B-cell leukemia/lymphoma 10 (Bcl10)	2013	Bhattacharyya et al.	23766559
Carboxymethylcellulose	Murine model	Bacterial overgrowth and increased bacterial adherence due to distention of spaces between villi and migration of bacteria to the bottom of the intestinal crypts	2009	Swidsinski et al.	18844217
Carrageenan	Murine model and peripheral blood monocytes	Increased inflammation levels and colitis linked to NF-κB activation.	2010	Benard et al.	20072622
Polysorbate 80	Murine model	Reduced microbiota diversity, colitis driven by elevated levels of lipopolysaccharide and flagellin in the metagenome patterns	2017	Viennois et al.	27821485
Carrageenan	Murine model	Inflammation promoted by LPS-induced IL-8 expression	2017	Wu et al.	28163398
Carrageenan	Murine model	Carrageenan-induced colitis linked with changes in gut microbiota's composition: a reduction of Akkermansia mucinofila, which has important anti-inflammatory properties	2017	Shang et al.	28778519
Glyceryl monolaurate	Murine model	Increased inflammation levels: upregulation of circulating levels of serum LPS, IL-1β, IL-6, and TNF-α. Dysbiosis in gut microbiota	2017	Jang et al.	29131494
Methylcellulose	Murine model	Methylcellulose exposure was associated with more severe colitis	2018	Llewellyn et al.	29174952
Polysorbate 80	Murine model	Increased ileal dysbiosis	2020	Furuhashi et al.	31359491
Polysorbate 80 and Carboxymethylcellulose	Murine model	Increased interleukin-1β expression, induced colitis, increased bacterial adherence, increased the Gammaproteobacteria abundance and decreased the α-diversity in the small intestine	2020	Viennois et al.	33027647
Carrageenan	Human intestinal epithelial cells	Increased inflammation levels (via activation of Bcl10 with NF-κB activation and upregulation of IL-8). Colitis.	2007	Borthakur et al.	17095757
Carrageenan	Human intestinal epithelial cells	Toll-like receptor 4 interaction induced the Bcl10-NFkappaB-interleukin-8 inflammatory pathway	2008	Bhattacharyya S et al.	18252714
Polysorbate 60 and Polysorbate 80	Caco-2 cell model	Alteration in intestinal permeability and increased bacterial translocation	2010	Roberts et al.	20813719
Carrageenan	Human intestinal epithelial cells	Alteration in intestinal permeability and increased bacterial translocation	2012	Choi et al.	22561171
Carrageenan	Caco-2 cell model	Inflammation due to increased secretion levels of TNF-α, IL-1β and IL-6; colitis	2013	Jiang et al.	24126493
Carrageenan	Caco-2 cell model	Alteration in intestinal permeability and increased bacterial translocation	2017	Fahoum et al.	27718308
Polysorbate 80 and Carboxymethylcellulose	Murine model and M-SHIME model	Alteration in microbiota composition	2017	Chassaing et al.	28325746
Polysorbate 80 and Carboxymethylcellulose	Porcine mucus model	Altered mucus production and bacterial colonization	2018	Lock et al.	29968743
Maltodestrin E1400, Polysorbate 80 and Carrageenan	Fecal samples collection	Alteration in microbiota composition, increased LPS levels and bacterial density	2021	Naimi et al.	33752754
Polystyrene nanoparticles	Murine model	Changing in microbial barrier: decrease Verrucomicrobiota genera and increase the abundance of Spirochaetota	2022	Xiao et al.	34715478
Polystyrene nanoparticles	Murine model	Production of ROS and oxidative stress in cells, activation of NF-κB/NLRP3 pathway, decrease in expression tight junction proteins (ZO-1, Claudin 1, and Occludin) levels, overexpression of inflammatory cytokines (TNF-α, IL-6, and IFN-γ)	2022	He et at.	35830933
Polystyrene nanoparticles	Murine model	Damage to the intestinal barrier due to increased reactive oxygen species (ROS)-mediated apoptosis of intestinal epithelial cells	2021	Liang et al.	34098985

AGEs are a heterogenous group of compounds which are formed during the Maillard reaction, a non-enzymatic reaction or glycation that occurs between reducing-sugars and free aminoacidic group of proteins, peptides or free amino acids. This process is very common in the food industry because it helps to improve taste, consistency, color and aroma (189). Many human and animal studies point out that AGEs could be partially absorbed in the intestinal lumen and could distribute across various tissues and organs. About 60% of absorbed AGEs were found after 3 days especially in kidney and liver, but they were also found in heart, lung and spleen (190, 191). AGEs could also be endogenously produced. An accumulation of endogenous and exogenous AGEs could increase the production of ROS and determine cellular dysfunction and apoptosis. In fact, these compounds can interact with surface receptors of the antigen presenting cells and with RAGE, the receptor for advanced glycation end products (192–194). An exposure to AGEs could determine in human enterocytes a sizable increase in the production of IL-25 e IL-33, thus shaping immune response to Th2-pathways and allergic inflammation (195). Furthermore, it has been demonstrated that AGEs also cause a downregulation of tight junction protein expression, with a higher mucosal permeability and higher susceptibility to environmental triggers, amplifying the inflammatory response and potentially induce also epigenetic modifications (196). This alteration can theoretically facilitate the development of FA due to an increased exposure to food antigens. In addition, AGEs can also increase oxidative stress, as we know that the binding of their cellular receptor (RAGE) on enterocyte's surface leads to the activation of an extracellular kinase pathway (ERK 1 and 2) and NF-kb pathway, which bring to ROS production (197).

AGEs could also have an extra impact on the development of food allergy. Heilmann et al. have shown in a murine study that the specific glycation of food antigens could have allergenic potential by influencing T-cell immunogenicity. Their study showed an increased production of several cytokines (IL-2, IL-17, IFN-y) with glycated-ovalbumin compared to native ovalbumin (198).

## The brain-gut-microbiome axis and its barrier

Most functions of GI physiology are influenced by neural control, both enteric nervous system (ENS) and autonomic nervous system (ANS). The influence on the gut is bidirectional, as the gut also sends information to the systems through complex pathways to achieve homeostasis, and alterations in this communication have been proven to be associated with diseases. Cross-communication between the brain and gut occurs through multiple biological axes involving the above-mentioned neural network, the neuroendocrine and immune system, and metabolic pathways, enabling bidirectional communication (199). The intestine physically communicates with the brain through 2 neuroanatomical pathways: the ANS and the vagus nerve (VN). The afferent VN innervates the mucosal and muscle layers of the gut, senses stimuli as luminal volume through mechanoreceptors or chemical stimuli (hormones, neurotransmitters, and metabolites) through chemoreceptors, and transmits these signals to the brain. On the other hand, the efferent VN transfers information from the central nervous system (CNS) to the gut. The other bidirectional exchange is guaranteed via the enteric nervous system (ENS) in the intestine, which is connected to the ANS and VN in the spinal cord and transmits information to the brainstem nuclei (200). Recently, the microbiome has emerged as an integral player in gut-brain communication, and the concept of a microbiome-gut-brain axis has substituted the previous gut-brain axis entity. Gut microbiota interplays with the human host in a mutually beneficial relationship, influencing the development and maturation of the immune, endocrine, and nervous systems. Microbiota composition depends on various factors including the physiological changes in the gastrointestinal tract such as motility and secretion, which are tightly regulated by the central nervous and enteric nervous systems (201). Changes in small intestine or large intestine motility, such as diarrhea or constipation, can cause dysbiosis (202). At the same time, several studies in germ-free animals have shown an abnormal gut motility and altered perception of inflammatory pain, stressing out the importance of microbiota in the regulation of gut motility (203, 204).

Moreover, the central nervous system can control microorganisms by affecting the secretion of serotonin, cytokines, catecholamines, and dynorphin from enteroendocrine cells, immune cells, and nerves, responsible for mucus production, secretory functions, mucosal immune responses, and direct changes in intestinal mucosal permeability (205). Despite the physical separation constituted by the intestinal epithelium, microbiota can also affect the brain-gut axis: the VN can sense microbial signals (i.e., bacterial metabolites) or can be influenced via microbiota-mediated modulation of enteroendocrine cells in the gut epithelium (206). Microbial metabolites include metabolites generated by the host and modified by the gut microbes, such as secondary bile acids, dietary product-derived molecules (compound K), or *de novo* synthesized compounds such as SCFAs. SCFAs may deeply affect gastrointestinal physiology, influencing peristalsis, visceral pain, epithelial proliferation, barrier function, host immunity, but also bacterial pathogenesis itself (207). SCFAs can epigenetically modulate epithelial cells; for example, butyrate enhances mucus production, activating the MUC2 promoter and enhancing histone acetylation in cell cultures. Moreover, SCFAs can bind to specific receptors like G- protein-coupled receptors (GPR), inducing the production of chemokines and cytokines. For example, butyrate induces the IL-18 excretion in epithelial cells by binding to GPR109a, thus protecting the colon against inflammation and carcinogenesis (208).

On the contrary, gut microbiota can also directly transmit signals to submucosal afferent nerve receptors which acts as microbe-associated molecular patterns (MAMPs) such as peptidoglycan or lipopolysaccharide (LPS) in the cell wall. MAMPs can activate various immune cells, especially innate immune cells such as macrophages, neutrophils, and dendritic cells. Inflammatory cytokines such as IL-1α, IL-1β, TNF-α, and IL-6 produced by these cells can pass through the blood-brain barrier and affect brain function by acting on receptors expressed on neurons and glial cells, especially microglia (209, 210). LPS can also directly affect the brain through the blood-brain barrier (211).

Gut microbiota can also influence the host's metabolic state and neuroendocrine system. For example, SCFAs produced by microbes regulate the expression and secretion of glucagon-like peptide-1 (GLP-1) through the free fatty acid receptor of L cells, controlling insulin release and appetite (212). In addition, secondary bile acids metabolized by microbes activate the G protein-coupled bile acid receptor (TGR5) of L cells to secrete peptide YY (PYY) and GLP-1 (213). PYY inhibits GI motility, slows food intake, decreases appetite, and increases energy consumption.

The intestinal microbiota can also affect the secretion of serotonin (5-HT), a neurotransmitter fundamental for different gastrointestinal functions, such as sensory-motor function and gut homeostasis. Specific spore-forming bacteria from both humans and mice can increase colonic and serum 5-HT levels in germ-free (GF) mice and ameliorate GF-associated gut dysmotility by producing SCFAs (214), which increase 5-HT production by enteroendocrine cells (EECs) (215, 216). Moreover, SCFAs can increase serotonin production by increasing the expression of tryptophan hydroxylase-1 in ECCs (217). In addition, indole produced by microorganisms metabolizing tryptophan can stimulate ECC receptors to promote serotonin secretion (218). Interestingly, the gut microbiota can directly produce neurotransmitters such as serotonin, dopamine, epinephrine, norepinephrine, γ-aminobutyric acid, and acetylcholine (219, 220). However, neurotransmitters have large molecular weights and cannot pass through the blood-brain barrier, making it difficult for neurotransmitters in the periphery to affect the central nervous system directly. It is possible that the gut microbiota can regulate and influence the metabolism or production of precursors that can pass through the blood-brain barrier (221). Some crosstalk pathways between brain and gut-microbiota are summarized in Table 3.

**Table 3:** Pathways of crosstalk between brain and gut-microbiota.

From gut microbiota to brain	From brain to gut microbiota
Immune system	Immune system
- MAMPs induce cytokines production (IL-1α, IL-1β, TNF-α, and IL-6) - SCFAs induce cytokines production and stabilize tight junctions	- Modulates mucosal immune system
Neuroendocrine system	Neuroendocrine system
- SCFAs induce 5-HT, GLP-1, PYY production	- Modulates 5-HT and catecholamines secretion - Modulates cortisol production - Modulates gut microbiota composition
Enteric nervous system	Enteric nervous system
- MAMPs can interact with submucosal afferent nerve receptors - Direct regulation of neurotransmitter precursors production (5-HT, dopamine, GABA, glutammate)	- Modulates dynorphin secretion
Circulatory system	
- MAMPs directly affect the brain through blood-brain barrier - Cytokines can modulate neurons and glial cells	
Vagus nerve	Vagus nerve
- Microbial metabolites direct action - Modulation from EECs in gut epithelium	- Interacts with gut through efferent fibers - Modulates catecholamines secretion - Modulates intestinal mucosal permeability

Microbial activity is fundamental in regulating epithelial permeability: indole derivatives, bile acid metabolites, conjugated fatty acids, polyamines, and polyphenolic derivatives are other microbial-derived compounds that actively regulate gut barrier function (222). In addition, gut microbiota as well as bacterial and viral infections can also influence intestinal barrier function through the modulation of tight junction expression and assembly (223, 224). In a Canadian study, germ-free mice proved to have a higher colonic expression of claudin-1 and occludin and a lower paracellular uptake of probes as compared with conventional mice, suggesting that commensal microbiota may control colonic tight junction proteins and paracellular permeability (225). The same study demonstrated that transplantation of fecal microbiota from healthy humans can restore the barrier features (paracellular permeability, colonic barrier structure) of conventional mice within a week. Altogether, these data suggest that gut microbiota is crucial in preserving the integrity of the intestinal barrier and preventing the systemic spread of potentially harmful antigens (226).

In particular, specific gut microbiota components may modulate the intestinal permeability differently. For example, colonization of germ-free mice with Bacteroides or Escherichia coli Nissle 1917 (EcN) led to the up-regulation of genes encoding for proteins such as small proline-rich protein-2 (sprr2a) and ZO-1 responsible for cellular adhesion (227, 228). Moreover, it has been demonstrated that the increase in paracellular permeability in patients with IBS could be ameliorated by EcN, thus reducing abdominal pain and bloating (229).

The gut microbiota might also overstimulate the immune system by facilitating a leaky gut, leading to the release of immune mediators, such as histamine, tryptase, serotonin, polyunsaturated fatty acids (12-hydroperoxyeicosatetraenoic acid, 15-hydroxyeicosatetraenoic acid, 5-hydroxyeicosatetraenoic acid, 5-oxoeicosatetraenoic acid and leukotriene B4), known to evoke sensory afferent over-stimulation and pain (230–232). Moreover, some recent studies confirmed that IBS is associated with reduced stability and biodiversity of the gut microbiota (233). Also, an Italian cross-sectional study revealed significant differences among IBS subtypes in the distribution of Clostridiales. Relative Clostridiales abundance was correlated with significant differences in the level of fecal SCFAs, which together were associated with altered fecal cytokine levels (234).

Similarly, in clear-cut inflammatory conditions such as IBD, microbiota dysbiosis has been well described in association with an impairment of intestinal permeability. The intestinal barrier may represent the target of mediators released by inflammatory cells in the lamina propria, and disruption of the physiologic barrier would then allow the passage of antigen, leading to further inflammation in a self-maintaining pathological inflammatory process and antagonizing tissue repair (235). In patients with IBD, there has been a loss of biodiversity (with a decreased concentration of Firmicutes species and Akkermansia muciniphila) and microbial stability, while an increased concentration of Proteobacteria such as Enterobacteriaceae, Bilophila, and Bacteroidetes (236, 237). Furthermore, in patients with IBD, a reduction in SCFA-producing bacteria such as *Faecalibacterium prausnitzii* has been observed (238). F. prausnitzii is well-known to have anti-inflammatory properties through its ability to produce butyrate, thus regulating T regulatory cell and T helper 17 (239). Doubtlessly, these changes may alter intestinal barrier integrity, potentially resulting in increased immune responses and the diffusion of pathogens into the intestinal tissues. The intestinal inflammatory process depends on the TLR pathway, responsible for the downstream production of cytokines such as TNF-α and IFN-γ, further fueling increased permeability both in IBD and IBS (240, 241).

## Conclusion

From the gathered evidence, it is clear that food antigens might interfere with gut permeability in diverse ways and in several diseases. This interference, however, does not rely on one-to-one relationship, given the fact that genetic background, barrier disruptors, microbiota perturbances and inflammation interact in a multidimensional fashion on a path towards disease onset. Unfavorable dietary choices and consequential changes in gut microbiota can promote intestinal barrier dysfunction and, subsequently, loss of tolerance towards food antigen and mucosal inflammation, with effects not only restricted to the gastrointestinal tract. On the other hand, inflammatory mediators can increase mucosal permeability and influence gut microbiota, leading to a vicious and self-maintaining circle.

Several gaps of knowledge still exist as regards this multi-level interaction. For example, it remains elusive why only one twin from monozygotic twins, with almost overlapping genetics, similar microbiota and diet, might develop a multifactorial disease, while the other twin might not. System biology approaches could hopefully sort out many unanswered questions if properly applied to a large set of metadata and biological samples from prospective cohorts.

By advancing our understanding of these intricate and interactive processes, future personalized therapies could more effectively alleviate the burden of food-triggered diseases and improve quality of life for affected individuals.
